# Biometric measurements of Santa Inês meat sheep reared on *Brachiaria brizantha* pastures in Northeast Brazil

**DOI:** 10.1371/journal.pone.0219343

**Published:** 2019-07-30

**Authors:** Joelma da Silva Souza, Gelson do Santos Difante, João Virgínio Emerenciano Neto, Ângela Maria Quintão Lana, Francisca Fernanda da Silva Roberto, Pedro Henrique Cavalcante Ribeiro

**Affiliations:** 1 Department of Animal Science, Federal University of Minas Gerais, Belo Horizonte, Minas Gerais, Brazil; 2 Department of Animal Science, Federal University of Mato Grosso do Sul, Campo Grande, Mato Grosso do Sul, Brazil; 3 Department of Animal Science, Federal University of Vale do São Francisco, Petrolina, Pernambuco, Brazil; 4 Department of Animal Science, Federal University of Paraíba, Areia, Paraíba, Brazil; 5 Department of Animal Science, Federal University of Rio Grande do Norte, Natal, Rio Grande do Norte, Brazil; Tokat Gaziosmanpasa University, TURKEY

## Abstract

This study was undertaken to examine biometric measurements during the growth phase of male and female Santa Inês sheep reared in *Brachiaria brizantha* pastures in northeastern Brazil. The experiment involved 24 castrated males and 24 females at an initial age of 90 days, with an average body weight of 19.04 ± 0.96 kg. Treatments consisted of the effect of four cultivars (Marandu, Xaraés, Piatã and Paiaguás) and two sexes. Six animals were used per treatment, in a randomized-block experimental design. The following characteristics were evaluated: abdominal circumference (AC), body condition score (BCS), body length (BL), body weight (BW), body capacity 1 (BC_1_), body capacity 2 (BC_2_), chest width (CW), heart girth (HG), leg circumference (LC), leg length (LL), rump height (RH), rump width (RW) and withers height (WH). Data were subjected to descriptive analysis, Pearson’s correlation, ANOVA and Tukey’s, Kruskal Wallis and Mann-Whitney tests. Univariate and multiple regressions were applied to estimate BW with a maximum error level of 5%. Significant differences were observed for the biometric measurements between sexes and cultivars (p<0.05). Body weight was highly correlated (>70%) with AC, WH, CG, RW, BC_1_ and BC_2_. The male sheep grazed on cultivars Piatã showed the best values for BW (40.43 kg), HG, RW, WH, LL, LC (102.46; 20.8; 65.23; 60.44; 42.54 cm respectively) and BC_1_ (4.25 kg/cm). Females grazed on cultivar Marandu had higher values for RW, CW, LL (17.26; 20.1; 75.98 cm respectively), BC_1_ (6.03 kg/cm) and BC_2_ (0.422 kg/cm). The equations that best estimated live weight were BC_1_ and HG. In male and female Santa Inês sheep, biometric parameters grow differently depending on the cultivar where they are grazed during the growth phase. Cultivars Marandu and Piatã are the most recommended for sheep production, as they provided the best performance and body development in those animals.

## Introduction

Sheep meat farming is a prominent activity in developing countries. This segment has a high revenue-generating potential in agribusiness. The demand for sheep meat grows annually, with the world production estimated to reach 23 million tons by the year 2020 [1]. In Brazil, meat and other products derived from sheep farming generated BRL 641.015 million in the year 2017 [2]. Sheep meat is composed of proteins of high nutritional value with chemical properties that are beneficial to human health [3]. However, its quality may be influenced by factors such as animal genotype, sex, weight, slaughter age and type of diet supplied.

Sheep production is characterized by diversities across production systems. The use of pasture-based systems with tropical forage grasses has yielded satisfactory results. The high biomass production per unit area and nutritional value of herbage, coupled with the low production cost, contribute to these results [4]. The better use of herbage produced in pasture-based systems influences the feeding behavior of animals, allowing for increased nutrient intake [5].

The management of a rearing system associated with factors inherent to the animal and environmental conditions have a direct impact on production indices [6]. Tropical grasses have been evaluated as a component of sheep diets in northeastern Brazil. In those studies, alterations were observed in the characteristics of those grasses over the year which ultimately influenced the production performance of grazing sheep [7]. In Brazil, the largest sheep flock is concentrated in the northeast region, with 9.03 million head [2]. Santa Inês is the most widespread sheep breed in that region. These animals stand out for their optimal meat production, hardiness, prolificacy, marked maternal ability and great ability to adapt to the climatic conditions of that region [8]. However, the efficiency of a production system and the quality of the supplied products depend, among other factors, on monitoring the growth and performance, slaughter age, and carcass yield of the farmed animals [9].

Biometric measurements are necessary in the evaluation of animal growth and performance and in determining the herd’s evolution in production systems. When analyzed together with morphological and physiological parameters, they constitute an important database for the evaluation of different sheep breeds in terms of genetics and nutrition [10]. Evaluating body measurements as a function of age in Santa Inês breed may help to better understand their growth and meat production potential [11].

The present study proposes to examine biometric measurements during the growth phase of male and female Santa Inês sheep reared in *Brachiaria brizantha* pastures in northeastern Brazil.

## Material and methods

**Fig 1:**
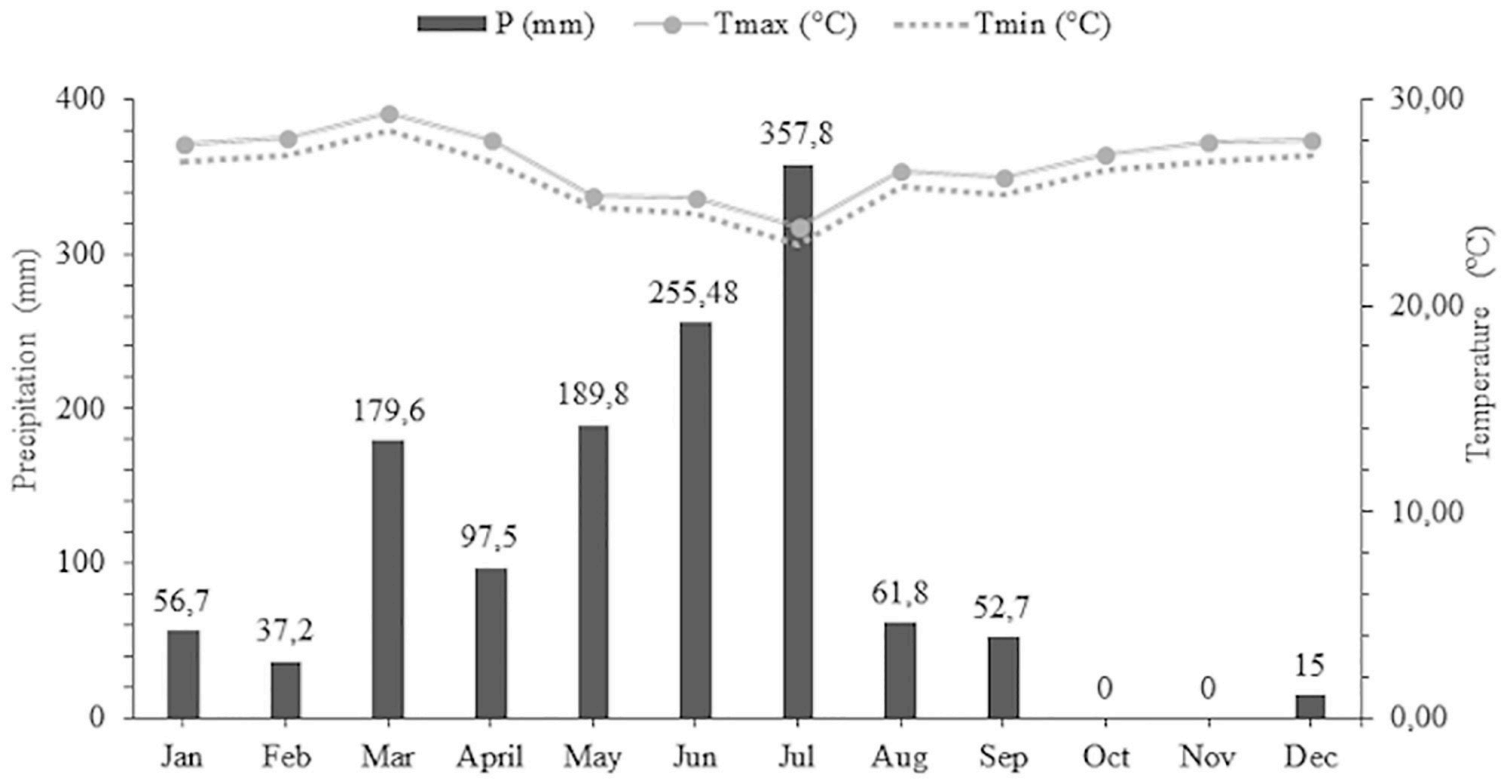
Precipitation and maximum-minimum temperatures during the experimental period.

The experiment was carried out at the Federal University of Rio Grande do Norte (UFRN), Macaíba Campus, located in Rio Grande do Norte, Brazil (5° 51' 30" S and 35° 21' 14" W; 11 m altitude). The climate of the region is a sub-humid dry type with rainfall surplus occurring from May to August (Thornthwaite, 1948). Average annual precipitation in the region is 1048 mm, and average annual cumulative potential evapotranspiration is 1472 mm. The temperature data of the experimental area were obtained from the database of the National Institute of Meteorology, while precipitation data were obtained using a *Ville de Paris*-type stainless-steel rain gauge installed in the experimental facilities (Fig 1).

The experimental area was a 2.88-ha field divided into eight 0.36-ha paddocks. Each plot was divided into six 0.06-ha paddocks, where the *Brachiaria brizantha* cultivars Marandu, Xaraés, Piatã, and Paiaguás were planted. Six paddocks were used per cultivar, under intermittent grazing. The pasture was managed for a pre-grazing height of 40 cm which is equivalent to 4592,86 kg DM and a post-grazing height of 20 cm which is equivalent to 2597,36 kg DM. The area was equipped with drinkers, salt troughs, and a rest area. The experimental period was 300 days, starting in March and ending in December 2017. All four *Brachiaria brizantha* cultivars (Marandu, Xaraés, Piatã, and Paiaguás) were planted in a randomized-block design with the fertility gradient considered in the block. The eight treatments were constituted by the four cultivars and two sexes, with six animals used per treatment (replicates). All procedures involving animals were approved by the Ethics Committee in Animal Experimentation at UFRN (approval no. 048/2016).

Twenty-four castrated male and 24 female lambs of the Santa Inês breed (initial age: 90 days; average live weight: 19.04 ± 0.96 kg) selected from the flock of the UFRN were distributed into groups that were grazed on one of the four cultivars. The animals remained in the pasture between 07h00 and 17h00, subsequently being moved to a shed near the experimental area during the night period in order to avoid being attacked by predators. To adjust the stocking rate, the herbage mass in the paddocks was estimated by harvesting six samples before grazing, using a 1-m^2^ frame where the grass was cut to a residual height of 20 cm. Pre-grazing forage availability was estimated by harvesting forage from six representative areas. The forage components were obtained by separating leaf, stem, and dead material and estimating their respective percentages (Table 1).

**Table 1:** Mean values of forage mass and percentage of structural components of *Brachiaria brizantha* cultivars in three grazing cycles.

Variable	Cultivar			
	Marandu	Xaraés	Piatã	Paiaguás
	Cycle 1			
Forage mass (kg/ha)	2643.84	2603.32	2191.61	2051.43
Leaf (%)	57.42	72.97	69.01	64.57
Stem (%)	30.81	19.20	24.39	26.72
Dead mass (%)	11.77	8.25	7.54	8.71
	Cycle 2			
Forage mass (kg/ha)	2800.28	2214.87	2357.36	2698.53
Leaf (%)	60.20	62.12	66.00	46.08
Stem (%)	19.86	26.18	25.10	23.68
Dead mass (%)	19.94	13.37	10.38	30.38
	Cycle 3			
Forage mass (kg/ha)	2674.24	2450.46	2861.96	2421.32
Leaf (%)	37.49	31.49	32.00	21.00
Stem (%)	23.31	23.99	28.00	20.80
Dead mass (%)	40.0	44.61	40.85	58.20

The animals received daily supplementation at the rate of 0.5% of their body weight (as-is basis), based on recommendations [12] for a daily gain of 150 g. The concentrate was composed of soybean meal, ground corn, livestock urea, and a mineral supplement (Ovinofós) with monensin. The chemical composition of concentrate and forages offered to the animals during the experimental period is described in Table 2. Body weight was measured every 30 days, using a digital scale, after a solid-feed deprivation period of 12 h. Afterwards, the animals were assessed for body condition score (BCS) [4] on a scale of 1 to 5 points, where 1 = very thin, 2 = thin, 3 = slightly fat, 4 = fat, and 5 = very fat.

**Table 2:** Chemical composition of forage cultivars and concentrate.

Feedstuff	Composition (% dry matter)				Concentrate	
	CP	NDF	ADF	LIG	Composition (as-is basis)	
Marandu grass	8.21	70.83	34.46	2.84		
Xaraés grass	8.31	71.23	43.26	2.94	Livestock urea	2.29%
Piatã grass	8.10	71.93	34.63	2.65		
Paiaguás grass	9.35	71.34	33.91	2.76		
Corn	8.09	35.18	7.21	3.7	*Mineral supplement (Ovinofós) with monensin	3.42%
Soybean meal	44.28	48.35	15.50	1.6		

Crude protein (CP), neutral detergent fiber (NDF), acid detergent fiber (ADF), and lignin (LIG).

*Composition (per kg of product): Na-147.0 g; Ca-120.0 g; P-87.0 g; S-18.0 g; Zn- 3800.0 mg; Fe-18000.0 mg; Mn-1300.0 mg; monensin sodium-1300.0 mg; F-870.0 mg; Cu-590.0 mg; Mo-300.0 mg; I-80.0 mg; Co-40.0 mg; Cr-20.0 mg; Se-15.0 mg.

Biometric measures were performed at 30-day intervals. After 270 days of age, only 50% of the test animals from each treatment were evaluated (six of each sex per cultivar). On that occasion, 24 animals were chosen at random to be removed from the experiment due to adjustments in stocking rate according to herbage availability in the paddocks, which was a consequence of low precipitation (Fig 1).

The biometric measures were taken using a tape measure and a typo meter with the animal standing in a proper vertical position. All measurements were taken from the left side of the animal, for uniformity purposes [13]. The following body measurements were performed: body length (BL)—distance between the cervicothoracic junction and the tail base at the first intercoccygeal joint; heart girth (HG)—external circumference of the thoracic cavity, under the armpits; abdominal circumference (AC)—obtained by measuring the abdominal cavity, passing the tape along the navel; chest width—distance between the lateral faces of the scapulohumeral articulation; rump width (RW)—distance between the greater trochanters of the femurs; withers height (WH); distance between the withers and the distal end of the forelimb; rump height (RH)—distance between the sacral tuberosity and the distal end of the hindlimb; leg length (LL)—distance between the greater trochanter of the femur and the border of the tarsometatarsal joint; and leg circumference (LC)—measured in the middle part of the leg, above the tibiofemoral-patellofemoral joint.

Body capacity, an objective measurement of *in vivo* conformation in sheep, was estimated by two indices: body capacity 1 (BC_1_), calculated as live weight (kg) divided by body length (cm); and body capacity 2 (BC_2_), determined as live weight (kg) divided by chest girth (cm).

The data were analyzed first by descriptive analysis, in which the mean, the standard error of the mean, and the confidence interval were calculated. Gaussian probability distribution and homoscedasticity of variance were evaluated by the Lilliefors and Cohran methods, respectively.

Analysis of variance (ANOVA) was performed by the generalized linear models (GLM) method, considering the effects of blocks, cultivars, sex, and age in the statistical model as fixed, according to the following model:

$$Yijkml=\begin{array}{cc} \mu +Bk+Ci+Sj+(C*S)ij+eijk+\text{Im}+\text{int}\;eracions+\alpha ijklm & \end{array}$$

where *Yijkml* = observation of animal l in block *k*, on cultivar *i*, of sex *j*, at age *m*; *μ* = overall mean; *Bk* = effect of block *k*; *Ci* = effect of cultivar *i* (Marandu, Xaraés, Piatã and Paiaguás); *Sj* = effect of sex *j* (ram or ewe); (*C* * *S*)*i* = interaction effect between cultivar and sex; *eijkl* = random error associated with each observation in the plot; Im = measure repeated over time in each animal; int *eracions* = all the possible interactions between the factors; and *αijklm* = random error attributed to the subplot.

Regression equations were estimated for animal age, observing the existing interactions in the result of ANOVA. The BCS variable was compared by the non-parametric Kruskal Wallis test for cultivars and Mann-Whitney for sexes [14].

Correlations were obtained by Pearson’s correlation analysis and the t test, considering significance at p<0.01. The criterion for the classification of the correlation coefficient was r ≥70% meaning a strong association and 30% <r≤70% indicating a moderate correlation [15].

Simple linear regression equations were used to estimate the functional relationship between the variables. Multiple regression was applied to determine which variables are capable of predicting live weight in rams and ewes, considering the biometric measurements evaluated in the study, in the model. Multiple regression was estimated by the Backward method. The criteria used in the choice of the equations were the coefficient of determination (R^2^) and the root mean squared error. The error rate assumed as a significant effect was a maximum probability level of 0.05.

## Results and discussion

The mean weights and body measurement values of the 270-day-old sheep were accompanied by low standard errors of the mean and confidence intervals. As a consequence, the means varied little (Table 3). The greatest variation in the data was observed in BW, whose coefficient of variation was 16.11%, indicating that this variable is the most susceptible to external influences such as climatic conditions. The body measurements showed coefficients of variation lower than 9%, which suggests that these measurements were not significantly influenced by environmental effects. Body weight and the BL, LW, RW, HG, and AC measurements showed similar means between the sexes, after a 270-day evaluation period.

**Table 3:** Descriptive analysis of the means (($\bar{x}$)), standard errors of the mean ($S(\bar{x})$), and confidence intervals (CI) of biometric variables in male (M) and female (F) Santa Inês lambs at 270 days of age, in Northeast Brazil.

Cultivar	Variable	Sex^1^	$\bar{x}$	$S(\bar{x})$	CI	Cultivar	Variable	Sex	$\bar{x}$	$S(\bar{x})$	CI
Marandu	Body weight (kg)	F	32.41a	1.33	28.98–35.84	Xaraés	Body weight (kg)	F	27.61a	0.98	25.07–30.15
		M	30.90a	3.80	21.12–40.67			M	29.93a	2.02	24.73–35.12
	Body length (cm)	F	57.21ab	0.56	55.77–58.65		Body length (cm)	F	54.06ab	0.80	52.00–56.12
		M	53.90ab	1.76	49.37–58.42			M	55.88ab	2.24	50.11–61.65
	Chest width (cm)	F	15.75a	0.30	15.74–15.75		Chest width (cm)	F	13.36a	0.55	13.94–16.79
		M	14.75a	1.12	11.86–17.63			M	16.16a	0.30	16.16–16.17
	Rump width (cm)	F	17.50a	0.56	16.05–18.94		Rump width (cm)	F	16.45a	0.38	16.44–16.45
		M	16.58a	0.89	14.27–18.89			M	18.00a	0.51	16.67–19.32
	Heart girth (cm)	F	92.16ab	1.44	88.44–95.88		Heart girth (cm)	F	87.00ab	0.96	84.51–89.48
		M	87.16ab	5.01	74.27–100.05			M	92.00ab	1.91	87.07–96.92
	Abdominal circumference (cm)	F	98.50ab	1.40	94.88–102.11		Abdominal circumference (cm)	F	92.50ab	1.78	87.91–97.08
		M	94.00ab	4.44	82.56–105.43			M	95.83ab	1.86	91.02–100.63
Piatã	Body weight (kg)	F	32.21a	1.26	28.96–35.46	Paiaguás	Body weight (kg)	F	27.96a	2.21	21.79–34.12
		M	34.16a	1.43	30.48–37.85			M	28.93a	1.91	24.01–33.85
	Body length (cm)	F	58.50a	1.51	54.60–62.39		Body length (cm)	F	52.84b	0.99	50.07–55.60
		M	57.25a	1.42	53.58–60.91			M	55.00b	1.82	50.30–59.69
	Chest width (cm)	F	16.03a	1.07	14.95–17.10		Chest width (cm)	F	15.00a	0.44	13.75–16.24
		M	15.91a	0.32	15.91–15.92			M	16.50a	0.34	16.49–16.50
	Rump width (cm)	F	17.66a	0.55	16.23–19.10		Rump width (cm)	F	17.60a	1.14	16.182–19.01
		M	18.00a	0.36	17.99–18.00			M	17.50a	0.34	17.49–17.50
	Heart girth (cm)	F	93.50a	2.59	86.83–100.16		Heart girth (cm)	F	84.00b	3.11	75.34–92.65
		M	95.16a	1.94	93.12–97.3			M	88.83b	1.95	83.80–93.86
	Abdominal circumference (cm)	F	97.33a	2.10	91.91–102.75		Abdominal circumference (cm)	F	92.60b	3.00	84.23–100.96
		M	100.50a	1.60	96.36–104.63			M	93.50b	1.68	89.16–97.83

^1^There was no significant difference between the sexes.

Means followed by different letters, within cultivars, differ according to Tukey’s test (p<0.05)

There was a cultivar effect (p<0.05) on the biometric measurements, with cultivar Piatã providing the highest BL, HG, and AC means for ewes and rams (58.50, 93.50, and 97.33 cm; and 57.25, 95.16, and 100.50 cm, respectively). Overall, Paiaguás grass provided lower biometric means than the three other cultivars, which may be explained by the lower percentage of leaves in that cultivar, as shown in Table 1. For BW, however, this statistical difference disappears due to the elevated dispersion of this variable, a characteristic inherent to body weight, according to the confidence interval. Therefore, using the variables least sensitive to external variables, with narrower confidence intervals, was essential in predicting animal body weight with greater efficiency. The observed uniformity of variables between both sexes is an indication that the animals had a homogeneous body size. This is an important fact for accurately determining biometric measurements, which serves as a premise in the formation of sheep lots in production systems. The mean values found in the present study corroborate results found in the literature for Santa Inês sheep in different age categories grazed on native and cultivated pastures, with and without supplementation [16]; on *B*. *brizantha* and *B*. *humidicola* [17]; and on Tifton 85 grass plus *Cnidoscolus quercifolius* forage salt [9].

The mean BW values of the animals at 270 days were similar to some reported by researchers [18] who evaluated 12-month-old Santa Inês sheep receiving different doses of concentrate while grazing on Tifton 85 grass. Those authors found BW means of 26.55, 28.55, 31.50, and 34.18 kg for the respective concentrate doses of 0, 0.66, 1.33, and 2.0%. The BW results obtained in the current study, in which a forage plant of lower nutritional value was employed, reveal the viability of farming meat sheep on *B*. *brizantha* cultivars in Northeast Brazil, a region whose main bottleneck in production is irregular herbage allowance throughout the year. Intensifying the use of those grasses can contribute to improving herd productivity and consequently elevating the profitability of production systems.

The Piatã, Marandu, and Xaraés cultivars provided a higher BL in rams and ewes, and the same was observed for the HG and AC measurements. The observed similarity in the development of these characteristics may be attributed to the morphological and structural conditions of the cultivars, whose herbage masses were equivalent in the three grazing cycles (Table 1). This might have had a positive effect on the growth potential expression of those animals. In young animals, growth rate is mostly related to feed intake, tissue growth capacity, and body maintenance efficiency [19]. Studies [20] with Santa Inês sheep on *B*. *brizantha* cvs. Marandu and Piatã and on *Panicum maximum* cvs. Massai and Aruana described BL values of 57.17, 55.58, 56.3, and 54.83 cm, respectively. These similar results may be associated with the genetics of the breed, the region where the experiment took place, and characteristics of the tested cultivars.

Leg width and rump width did not differ between the sexes or cultivars, which is probably related to the BW values, since these measurements are directly influenced by the nutritional status of the animal and by its development stage. The mean values of those measurements are within the observed range reported in studies [21, 22] with those animals, where authors found 15.53 and 16.55 cm for chest width and 13.52 and 15.8 cm for rump width, respectively. The average HG and AC of the rams and ewes in the current study were higher than those found [20] in uncastrated rams of the same breed with a live weight of 30 kg grazing on Marandu, Piatã, Aruana, and Massai pastures (82.12, 75.17, 83.92, and 81.83 cm; and 81.42, 82.33, 83.75, and 81.08 cm, respectively). This superiority is likely related to external effects, which include herbage allowance, nutritional value, parasitic frequency, and animal genotype. In an experiment on the production performance of six-month-old Santa Inês sheep [17], researchers obtained an average HG of 85.86 cm. As HG increases, so does the animal’s weight gain ability, due to its greater respiratory capacity and dry matter intake [22], which may be considered a good indicator of BW.

**Fig 2:**
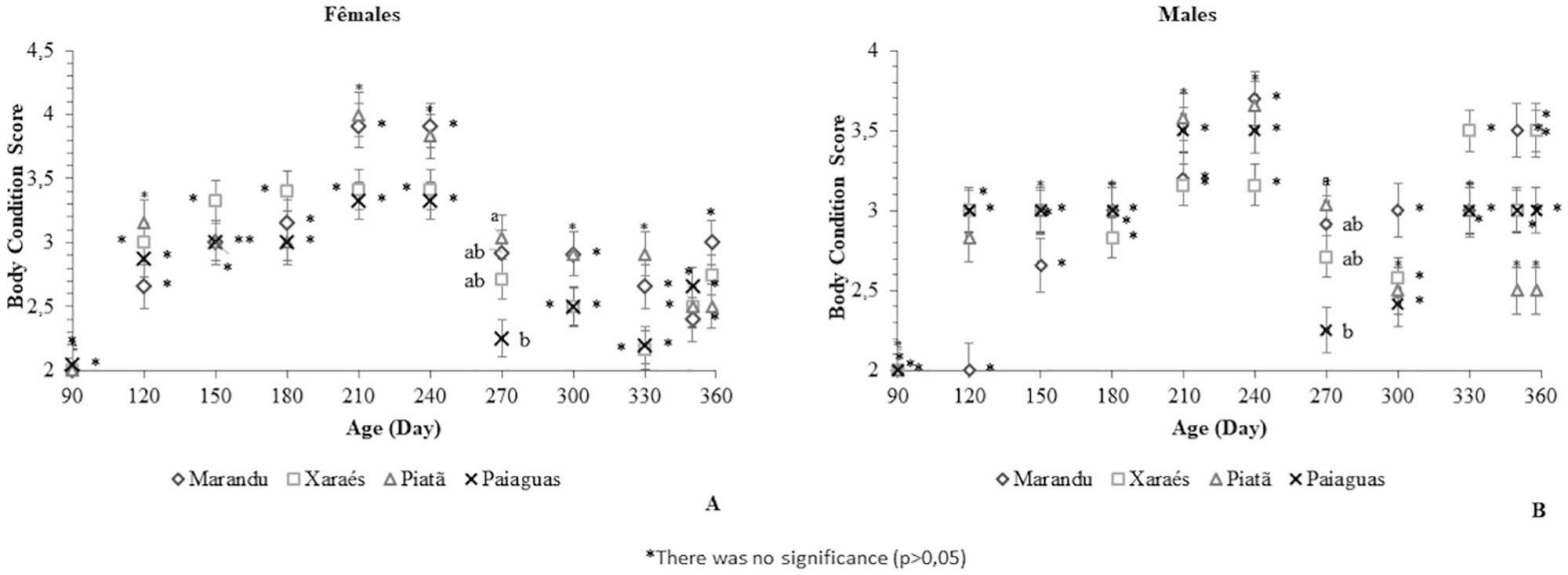
Body condition scores of Santa Inês sheep in an intermittent grazing system on different *B*. *brizantha* cultivars in Northeast Brazil.

In the analysis of BCS, whose purpose is to assess the degree of fat deposition in the animal carcass, there was no interaction effect between sex and cultivar or an effect of sex alone (p>0.05). Age, however, influenced this variable (p<0.05). Regardless of sex, the cultivar that provided the highest BCS at 270 days was Piatã (BCS = 3.04). The sheep on Paiaguás pasture had the lowest BCS, 2.42 points (Fig 2A and 2B). Scores greater than 3 indicate slightly heavier animals with little fat deposition in the carcass [23]. The animals on Paiaguás grass showed inferior BCS, despite their response for BW, which may be related to the lower leaf allowance, as shown in Table 1. The positive result obtained with the Piatã cultivar is likely a consequence of the higher herbage mass production observed in that period (approximately 2861.96 kg/ha) coupled with the 32% leaf percentage and lower dead mass percentage (40.85%), which led the animals to eat more.

The BCS of rams and ewes on all cultivars decreased gradually after 270 days of age. This decrease was likely due to the low herbage production observed in the last grazing cycle (Table 1), which, in turn, was a consequence of the low precipitation occurring in the period (Fig 1). Results for BCS may be considered a basic tool to monitor the weight development of animals in a production system.

The following measurements were highly associated (>70%) with BW: AC (r = 0.82), BL (r = 0.81); WH (r = 0.79), HG (r = 0.78), RH (r = 0.73), LC (r = 0.70), RW (r = 0.70), BC_1_ (r = 0.76), and BC_2_ (0.82). These high correlations indicate elevated meat production capacity, and these measurements can thus be used as selection tools [6, 15, 17, 24], as they are highly and positively correlated with animal weight [25]. Positive correlations were also found between BC_2_ and biometric measurements in ½ Santa Inês + ½ Dorper and ½ Suffolk + ½ mixed-breed sheep [26]. The LC, LL, LW, and RW measurements showed lower *r* values than the others; therefore, they provide little accuracy in estimating BW when compared with highly related measurements.

In the trunk region, BL was strongly associated (≥0.70) with WH (r = 0.75), HG (r = 0.70), AC (r = 0.72), and BC_2_ (r = 0.77), which is likely due to the homogenous body growth among the animals. Two other highly associated measurements were WH and RH (r = 0.86). This correlation is important in determining animal body size, since the bone base is the main aspect considered during these measurements [27]. To However, this true for the other measurements, as they are also influenced by muscle and fat [13].

**Fig 3:**
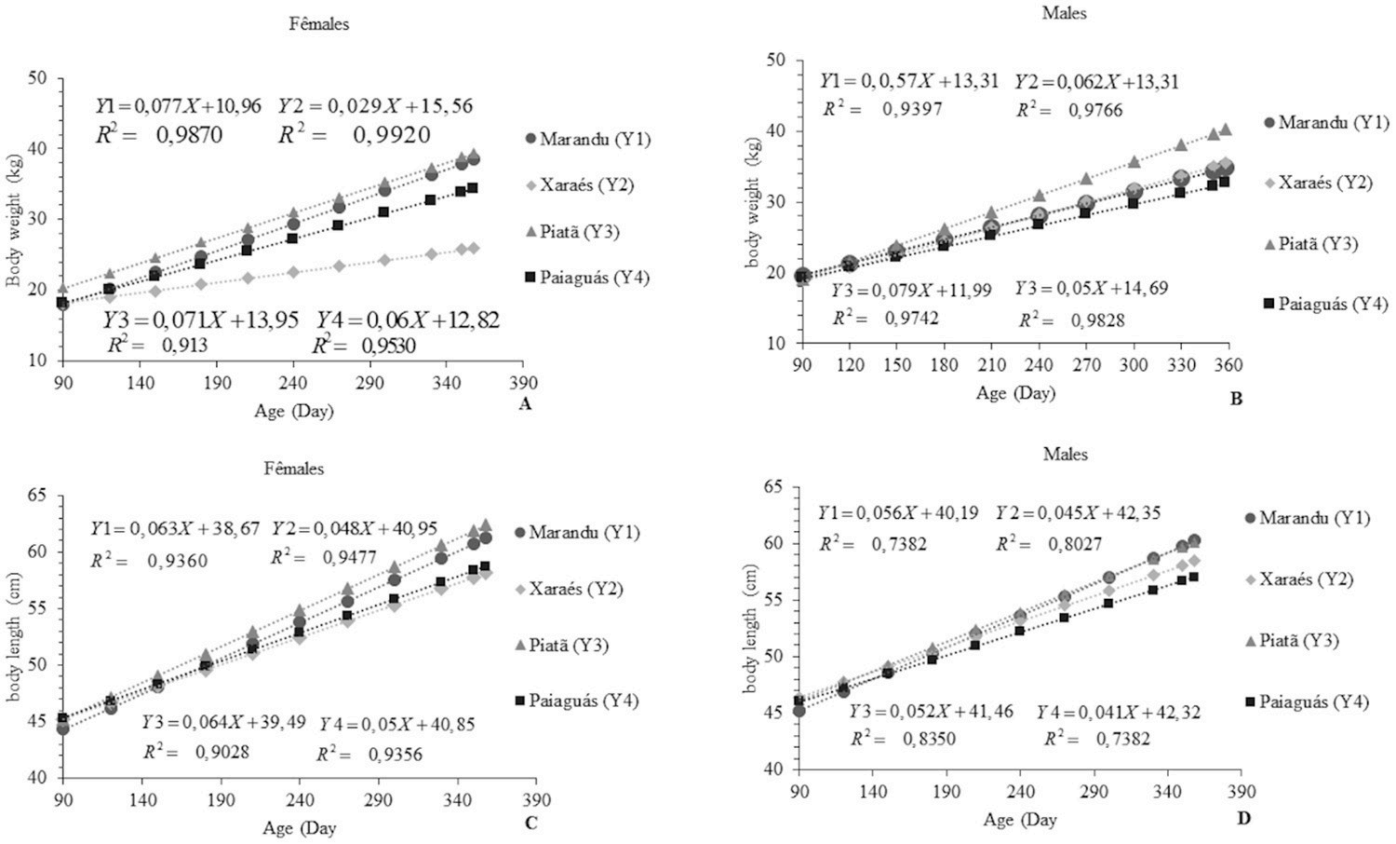
Linear behavior of body weight (A and B) and body length (C and D) as a function of age in Santa Inês rams and ewes on *Brachiaria brizantha* cultivars.

There was a significant interaction effect between cultivar, sex, and age (p<0.05) in the regression analyses. The highest BW gain for both rams and ewes was obtained with cultivar Piatã (average: 39.51 and 40.43 kg, respectively; (Fig 3A and 3B). This better performance is possibly a result of the compensation between produced and consumed forage, since there were no significant differences in nutritional value across the cultivars (Table 2). Piatã has the thinnest stem among the cultivars, which might have favored intake [28]. Variations in the grazing process arising from changes in the canopy structure may influence forage intake and, consequently, animal production [29].

The rams kept on Piatã and Xaraés pastures gained more BW than the ewes (53.23 vs. 10.13%, respectively). In the Marandu and Paiaguás cultivars, the females were heavier than the males (25.98 vs. 16.67%, respectively). Weight gain usually responds differently according to sex, with males gaining more due to differences in sex hormones [30]. This behavior was distinct across the cultivars, possibly because the effect of sex was not significant to the point of causing greater BW only for the rams. This effect is likely more noticeable as the animals reach adult age [16].

The weight gain of the sheep on Marandu, Xaraés, Piatã, and Paiaguás pastures over the 360-day period was 34.98, 35.63, 40.43, and 32.69 kg for rams and 38.68, 26, 39.51, and 34.42 kg for ewes, respectively. These values correspond to 43.72, 44.53, 50.53, and 40.86% of the weight of an adult Santa Inês ram and 64.46, 43.33, 65.85, and 57.36% of the weight of an adult Santa Inês ewe, in the respective order of cultivars. These results demonstrate the viability of farming meat sheep using those cultivars. Studies involving Santa Inês rams and ewes also reported that these animals reached 70% of adult weight at 360 to 450 days of age [31]. The current results agree with the statement that Santa Inês is for meat production. However, achieving this production requires excellent animal management control, high-quality diets, and technologies that indicate the ideal moment for slaughter.

Ewes on the Piatã pasture showed the best results for BL, growing 0.064 cm per day, which resulted in an average BL of 62.53 cm. The rams with the highest BL were those which grazed on Marandu grass, growing 0.056 cm per day and averaging a BL of 60.19 cm at 360 days (Fig 3A and 3B). A comparison between the effects of the Marandu, Xaraés, Piatã, and Paiaguás cultivars on the sexes showed that the females had longer bodies than the males on all cultivars (approximately 88.88, 93.75, 81.25, and 82%, respectively). This trend is possibly related to hormonal action, which influences tissue growth in each sex differently.

A similar study with Santa Inês, Dorper × Santa Inês, and Dorper × Morada Nova sheep revealed a different behavior from that observed in our study, with no sexual dimorphism occurring in any of the traits evaluated from birth to 180 days of age [11]. In the present study, the animals already exhibited sexual dimorphism at 180 days. Regardless of the sex, the present values are similar to the average BL of 62.02 and 65.95 cm reported in performance trials with Santa Inês sheep [11, 32]. Body length values indicate loin length, and when associated with height and weight, they can reveal the carcass conformation of an animal after slaughter.

**Fig 4:**
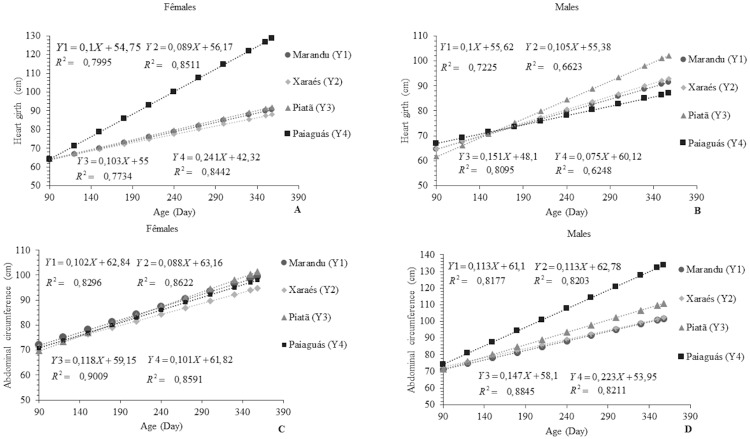
Linear behavior of heart girth (A and B) and abdominal circumference (C and D) as a function of age in Santa Inês rams and ewes on pastures of *Brachiaria brizantha* cultivars.

Heart girth is a measure that indicates the potential of an animal for meat production [17]. Ewes on Paiaguás pasture showed the highest value for this variable 129.08 cm, which corresponds to 63.08, 58.51, and 57.24% of those observed in the animals on the Xaraés, Marandu, and Piatã pastures, respectively. The rams with the highest HG were those which grazed on Piatã pasture, averaging 102.46 cm. This value was 50.34, 33.78, and 30.47% higher than those shown by the rams on the Paiaguás, Marandu, and Xaraés, respectively (Fig 4C and 4D).

The results obtained for the Paiaguás and Piatã cultivars are probably related to the structural characteristics of those forages such as thinner stems, and in the case of Paiaguás, smaller leaves [28]. This fact might have led to a higher intake of forage mass by the animals. The average HG values found in the above-mentioned study corroborate the present findings for rams and ewes (124.28 and 114.97 cm, respectively). In the analysis of cultivar effect on HG in rams and ewes, a difference was observed for HG between the animals on Piatã, Xaraés, and Paiaguás pastures. Males grew approximately 31.79 and 15.29%, respectively, in relation to the females on Piatã and Xaraés pastures. The ewes, in turn, grew 68.88% in relation to the rams on the Paiaguás pasture.

The opposite of what occurred for HG was observed for AC, with the ewes on Piatã grass showing the highest AC (average: 101.63 cm). Males on Piaguás pasture showed an AC of 134.23 cm. These results were possibly influenced by the same effects observed for HG in those animals. A sex effect (p<0.05) was detected for the animals on Paiaguás, Xaraés, and Piatã pastures, with rams showing a 54.7, 20.0, and 19.73% larger AC than the ewes, respectively. The males possibly had a faster bone growth that was caused by the action of hormones [21], which is translated into larger dimensions compared to females in the same period.

**Fig 5:**
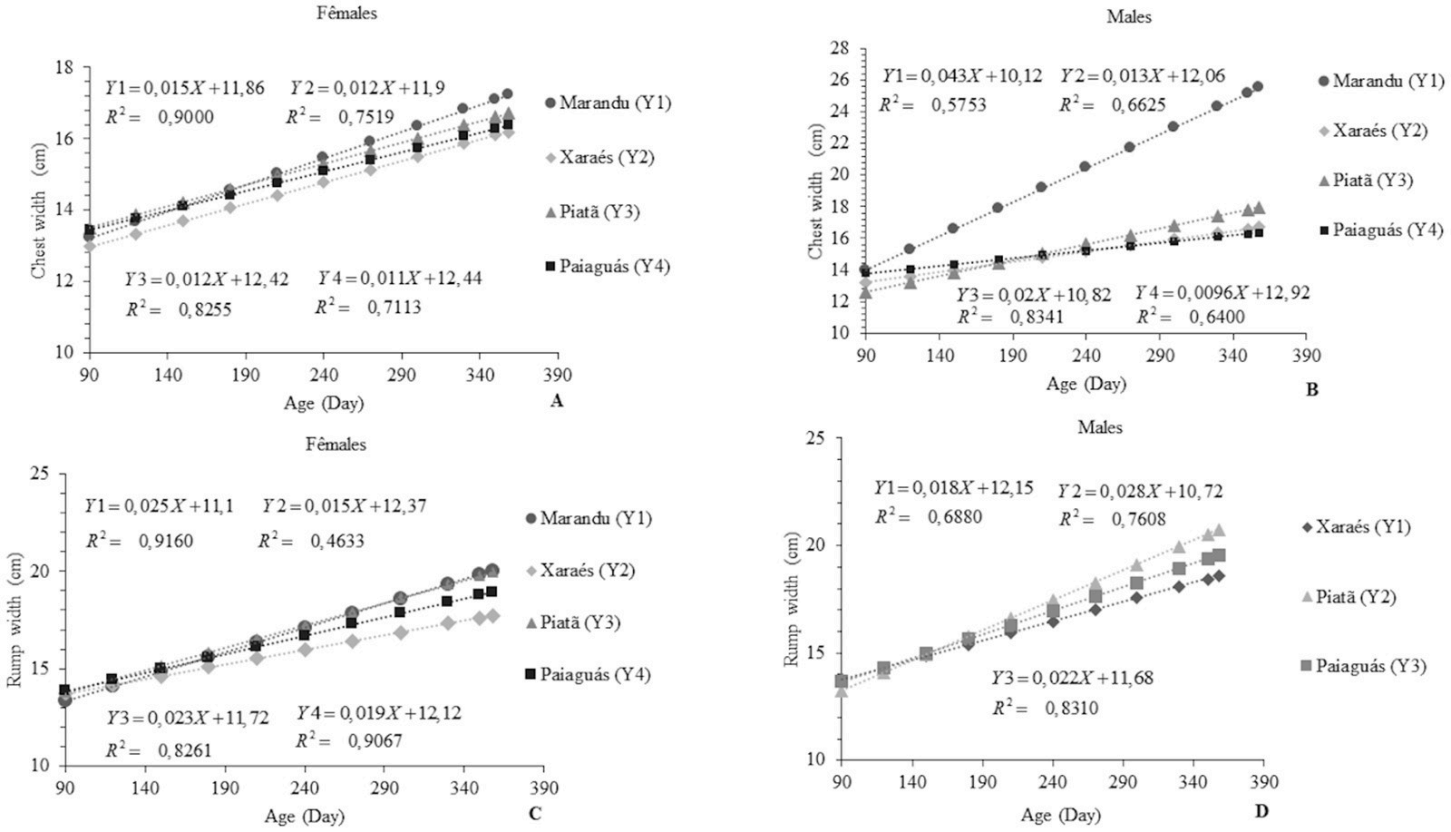
Linear response of chest width (A and B) and rump width (C and D) as a function of age in Santa Inês rams and ewes on pastures of *Brachiaria brizantha* cultivars.

The LW and RW measurements were also influenced by the cultivars (p<0.05) (Fig 5A and 5B). In rams and ewes, the highest LW was observed for those grazing on Marandu pasture (average: 25.6 and 17.26 cm, respectively). For this cultivar, the ewes showed a higher RW than the rams, averaging 20.1 cm. The rams on Piatã grass showed the highest RW: 20.8 cm. For this variable, the Marandu cultivar did not show function fitting. The observed results may be a consequence of the BW of those animals on the same cultivars. The LW values agree with those reported in studies [20] with Santa Inês sheep on Marandu, Piatã, Massai, and Aruana pastures, where the authors obtained mean LW of 18.75, 17.5, 17.08, and 16.82 cm, respectively. The results for RW in the current study were higher than the 15.41 and 15.0 cm found in an experiment [20] evaluating animals grazing on Marandu and Piatã pastures, respectively. This may be related to the greater growth of the animals in the present study. A higher RW may provide increased muscle proportion in the leg, which is a desirable trait in meat sheep.

For CW and RW measurements, the sex effect was also expressed, with the rams showing a 65.12% and 40% higher LW than the ewes on Marandu and Piatã pastures, respectively. This effect was also observed for RW (Fig 5C and 5D), for which the males on Piatã, Xaraés, and Paiaguás pastures were superior to the females on all cultivars by 17.86, 11.67, and 13.64%, respectively. The BW of those animals might have contributed to those results, since those measurements are directly related to nutritional performance. These cultivars possibly caused distinguished effects on the muscle development in the animals. It is known that the type of diet can influence muscle development and composition in an animal [33].

**Fig 6:**
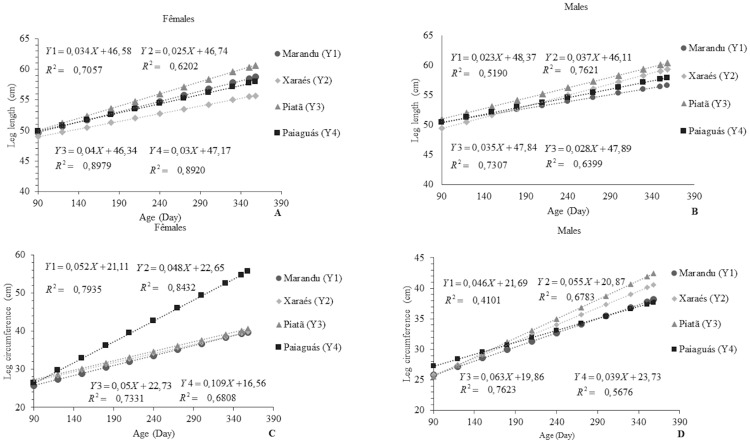
Linear behavior of withers height (A and B) and rump height (C and D) according to age in Santa Inês ewes and rams on *Brachiaria brizantha* cultivars.

Ewes on the Piatã pasture showed a WH of 66.68 cm, which is 64.29, 53.39, and 25% higher than the WH of the ewes grazing on the Paiaguás, Marandu, and Xaraés cultivars, respectively (Fig 6A and 6B). Among the rams, those with the highest WH grazed on Paiaguás pasture (average: 70.82 cm). This growth was 68.92, 56.76, and 33.79% greater than those shown by the rams on Marandu, Xaraés, and Piatã pastures, respectively. There was a sex effect for this variable, with the ewes on Xaraés and Piatã grasses having a 49.21 and 41.67% higher WH than the males, respectively. The rams on Paiaguás and Marandu pastures were superior to the females by 59.46 and 42.5%, respectively, for this variable. The present WH values are within the range observed in studies with Santa Inês sheep fed diets based on grass hay and concentrate containing sunflower cake (WH = 64.8 cm) [22].

Cultivar and sex effects were observed for RH (p<0.05). Cultivar Marandu provided the highest RH in the ewes (average: 75.98 cm). This growth was 67.05 and 11.37% higher compared with that achieved on the Paiaguás and Piatã cultivars. This result may explain the higher RW observed in the females grazed on Marandu pasture. In the rams, cultivar Piatã provided the highest RH, 65.23 cm. This value corresponds to 59.54, 41.87, and 23.26% of the RH obtained by the animals on Marandu, Paiaguás, and Xaraés pastures. Between the sexes, the females those with a 70.46, 60.25, 44.88, and 13.8% higher RH than the males. Results for WH and RH indicate leggy animals with likely well-developed shoulders and thighs.

**Fig 7:**
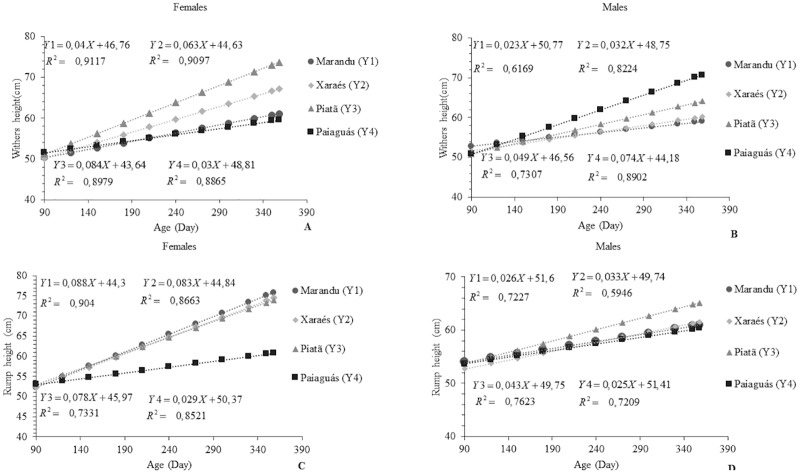
Linear behavior of leg length (A and B) and leg circumference (C and D) according to age in Santa Inês ewes and rams on *Brachiaria brizantha* cultivars.

The females with the highest LL (58.82 cm) were those grazing on Marandu pasture (Fig 7A and 7B). The males with the highest LL (60.44 cm), in turn, were those which grazed on Piatã grass. The highest LC in the Piatã pasture was obtained by the males: 42.54 cm. In the case of females, the highest LC (55.8 cm) was observed in the animals on Paiaguás grass. Considering these responses, it can be inferred that long legged animals are not always those with the highest LL and LC.

Ewes had a higher LL than rams on the Marandu and Piatã grasses (32.36 and 12.5%, respectively). Differences in LL and LC growth between males and females were detected in the groups on Xaraés cultivar (32.44 vs. 12.73%, respectively). The LC of ewes on Paiaguás, Piatã, and Marandu pastures was 64.23, 20.64, and 11.54% more developed than in the rams (Fig 7C and 7D). Overall, the ewes showed greater muscle mass in the leg, which indicates better meat cuts. The leg meat may account for up to 50% of the carcass weight of sheep slaughtered at 30 kg [33].

**Fig 8:**
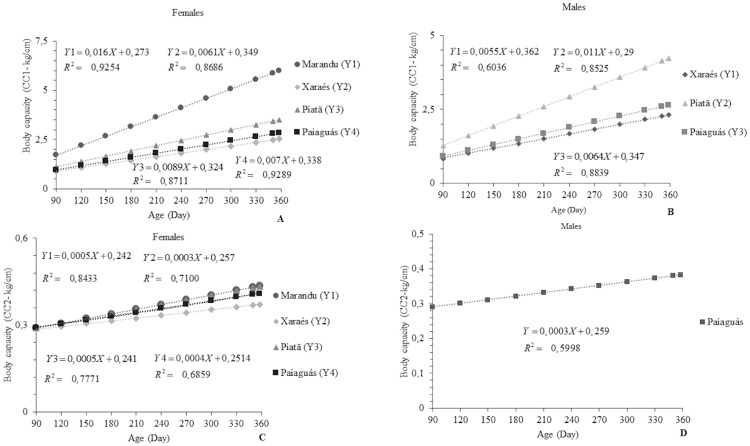
Body capacity indices (BC_1_ and BC_2_) according to age in Santa Inês ewes (A and B) and rams (C and D) on *Brachiaria brizantha* cultivars.

Body capacity indices BC_1_ and BC_2_ responded linearly (p<0.05). However, cultivar Marandu did not fit any functions for BC_1_, and neither did BC_2_ in cultivars Marandu, Xaraés, and Piatã (Fig 8A, 8B, 8C and 8D). The ewes with the highest BC_1_ were those on the Marandu pasture, averaging 6.03 kg/cm. This finding may be due to the higher BW observed in the group grazing on Marandu pasture. The rams with the highest BC_1_ were those grazing on Piatã grass, averaging 4.25 kg/cm. BC_1_ indices greater than 1.0 kg/cm indicate animals with low conformation, which is a desirable trait in meat animals [34].

For BC_2_, the females on Marandu grass and the males on the Paiaguás pasture showed the best results: 0.422 and 0.367 kg/cm, respectively. This index increased gradually as the animals grew older. The increasing values indicate increased deposition of muscle and adipose tissue per unit of growth, showing that the animals have potential for meat production. Similar results were reported in studies [27] investigating the same indices in Santa Inês rams and ewes aged 360 to 540 days. The observed BC_1_ were higher than 1.0 kg/cm, whereas BC_2_ ranged between 0.50 and 1.0 kg/cm in both sexes. In rams and ewes, BC_1_ was higher than BC_2_, and this response likely has a direct relationship with the growth speed of the BW, BL, and HG measurements. Body length growth is stabilized as bone growth stops, while BW and HG may continue growing even if bone growth is stabilized, because these measurements are more closely related to animal age and nutritional conditions, which depend directly on the type of diet supplied to them [16].

As shown in Table 4, the live weight of ewes and rams can be explained by the BC_2_ index and HG. The main advantage of using the body compactness index and heart girth in predicting body weight is increased measuring reliability; i.e., these variables are less sensitive to external factors. A study [35] involving similar conditions evaluating the performance of Santa Inês lambs showed that the equation that best explained live weight was heart girth. Studies with lactating Santa Inês sheep reared in the Amazon showed that the best equation to predict live weight in sheep until 60 days of age was composed of the following variables: body length, rump height, heart girth, rump width, and leg circumference [17]. In the present study, although these measurements were significant, they were accompanied by an R^2^ ≤ 0.99% and because this was the criterion determined for the equation, it was not possible to consider these measurements in the estimate of BW.

**Table 4:** Regression equations to estimate the live weight of Santa Inês rams and lambs on *Brachiaria brizantha* cultivars according to biometric measurements.

Cultivar	Sex	Prediction equation	Adjusted R^2^ (%)	P
Marandu	Female	BW = –26.35 + 78.45 BC_2_ + 0.34 HG	99.30	0.00001
	Male	BW = –24.34 + 73.34 BC_2_ + 0.34 HG	99.03	
Xaraés	Female	BW = –24.70 + 75.90 BC_2_ + 0.33 HG	99.47	0.00001
	Male	BW = –22.97 + 73.26 BC_2_ + 0.32 HG	99.24	
Piatã	Female	BW = –27.07 + 80.22 BC_2_ + 0.34 HG	99.37	0.00001
	Male	BW = –24.98 + 73.88 BC_2_ + 0.34 HG	99.46	
Paiaguás	Female	BW = –26.94 + 77.81 BC_2_ + 0.35 HG	99.57	0.00001
	Male	BW = –23.76 + 72.96 BC_2_ + 0.32 HG	99.50	

BW = body weight; BC_2_ = body capacity index 2 = live weight (kg)/heart girth (cm); HG = heart girth; P = error probability; R^2^ = coefficient of determination.

The estimated equations can accurately predict the live weight of Santa Inês sheep aged up to 12 months in the present production conditions. Body capacity index 2 explained 78.2, 75, 74.5, and 79% of the live weight of ewes and 86, 81.33, 68.87, and 78.45% of the live weight of rams on Marandu, Xaraés, Piatã, and Paiaguás pastures, respectively. The higher percentage observed for the males is likely due to the higher HG of those animals, which is influenced by the bone base and muscle and fat tissues, as a function of weight.

Researchers [32] evaluating the weight and yield of carcass and non-carcass components of Santa Inês sheep with an average live weight of 32 kg, at 120 days of age, concluded that the equation to estimate cold carcass weight as a function of heart girth showed the best fit and considered it practical for use in production conditions. The adoption of easily executed and low-cost biometric measurements and equations capable of accurately predicting live weight are important tools for sheep farmers, allowing them to estimate the animals’ live weight and the productive capacity of their carcass.

## Conclusions

The Santa Inês rams and ewes showed distinct growth of biometric measurements across cultivars, during the growth phase. Marandu and Piatã cultivars are the most suitable for sheep farming, as they allow for improved production performance and body development.

## Supporting information

S1 File Biometric measurements of Santa Inês meat sheep in Nordeste of Brazil. (XLSX)
